# Long non-coding RNA *AC087388.1* as a novel biomarker in colorectal cancer

**DOI:** 10.1186/s12885-022-09282-0

**Published:** 2022-02-21

**Authors:** Arash Poursheikhani, Mohammad Reza Abbaszadegan, Mohammad Amin Kerachian

**Affiliations:** 1 Medical Genetics Research Center, Mashhad University of Medical Sciences, Mashhad, Iran; 2 Department of Medical Genetics, Faculty of Medicine, Mashhad University of Medical Sciences, Mashhad, Iran; 3 Immunology Research Center, Mashhad University of Medical Sciences, Mashhad, Iran; 4 Cancer Genetics Research Unit, Reza Radiotherapy and Oncology Center, Mashhad, Iran

**Keywords:** LncRNA, Long non-coding RNA, AC087388.1, Biomarker, Colorectal Cancer, CRC

## Abstract

**Background:**

Several investigations have reported diverse roles of long non-coding RNA (lncRNA) in biological processes, tumor development, and progression of colorectal cancer (CRC). In this study, we investigated the lncRNA *AC087388.1* tumorigenic role in CRC cells.

**Methods:**

The CRC tissues were collected at the Reza Radiotherapy and Oncology Center, Mashhad, Iran. The human SW-48 and HT-29 CRC cell lines were obtained from the national cell bank of Iran. The cells were cultured according to ATCC (the American Type Culture Collection) recommendations. Quantitative real-time PCR was applied to assess the RNA expression. ShRNA transfection was done to downregulate the target gene. MTT and apoptosis assays were conducted to evaluate cell proliferation and viability, respectively. Colony formation assay, wound healing assay, and invasion assay were applied to determine growth, motility, and invasion of the cells, respectively. ENCORI online tool was used as downstream enrichment analysis.

**Results:**

Forty CRC patients were encompassed in this study. The results demonstrated that the lncRNA *SLC16A1-AS1, AC087388.1*, and *ELFN1-AS1* were significantly overexpressed in the CRC tissues in comparison to their normal counterpart margins. All the lncRNAs have shown significant Area Under Curve (AUC) values in the patients. Downregulation of lncRNA *AC087388.1* remarkably decreased the cell proliferation and viability of the CRC cells. In addition, the data demonstrated that the downregulation of lncRNA *AC087388.1* significantly suppressed cell growth and colony formation capability in the cells. Also, downregulation of lncRNA *AC087388.1* attenuated motility and invasion of CRC cells, and significantly decreased the expression of invasion genes. In-silico functional enrichment analysis indicated that the lncRNA *AC087388.1* has contributed to crucial signaling pathways in tumorigenesis such as the p53 and Wnt signaling pathways, apoptosis, and cell cycle.

**Conclusions:**

Altogether, we showed that lncRNA *AC087388.1* has an oncogenic role in tumorigenesis of CRC, and it can be considered as a novel diagnostic and prognostic biomarker in CRC.

**Supplementary Information:**

The online version contains supplementary material available at 10.1186/s12885-022-09282-0.

## Background

Colorectal cancer (CRC) is one of the most frequent malignancies of gastrointestinal (GI) system and according to GLOBOCAN 2020, it accounts for the fifth leading cause of cancer-related death, globally [1, 2]. It has been demonstrated that CRC tumorigenesis is correlated to different kinds of genetic and epigenetic variations, and its development is a complex multi-step biological process [3–5]. However, the clear mechanism of CRC tumorigenesis is not completely understood yet.

The gold standard method for screening CRC patients is colonoscopy which combines diagnosis, and treatment, but it is an invasive approach. There are other screening methods such as guaiac fecal occult blood test (gFOBT), and fecal immunochemical test (FIT) which have a lack of sensitivity and specificity [6]. Due to the lack of early and precise diagnosis, and distance metastasis of CRC, the majority of CRC patients are diagnosed in the advanced stages with poor prognoses [7, 8].

In recent years, despite improving the CRC treatment approaches such as surgical resection, radiation, and chemotherapy, unfortunately, the 5-year survival rate of the patients is disappointing (less than 30%) [9, 10]. Therefore, there is an urgent need to discover and develop an efficient diagnostic and prognostic biomarker for CRC.

Recently, a large body of investigations reported diverse roles of non-coding RNA, particularly long non-coding RNA (lncRNA), in biological processes of different sorts of cancer [11–13]. LncRNAs are a group of non-coding RNAs with more than 200 nt in length and with no or little capability of coding proteins [14–16]. They have been demonstrated to play different canonical roles in diverse biological processes such as cell proliferation, differentiation, and cellular development, carcinogenesis, and metastasis through regulating cornerstone genes expression [4, 17]. Numerous investigations highlighted the crucial role of lncRNAs in cancer development and progression [18]. For instance, it has been demonstrated that lncRNA cCSC1 induced self-renewal capacity and drug resistance (stemness characteristics) in CRC cells through recruiting Hedgehog signaling pathway [19]. In another example, lncRNA *SNHG16* has been illustrated to regulate cell proliferation, invasion and metastasis by upregulating *MCP1* expression through sponging miR-124-3p in CRC cells [20]. lncRNA *MALAT1* has been shown to induce resistance to irradiation in CRC cells via inhibiting miR-101-3p [21]. Altogether, the previous investigations proposed that lncRNAs can be considered as novel therapeutic targets and desired biomarkers in CRC.

According to our previous study [4], we comprehensively demonstrated lncRNA-miRNA-mRNA regulatory networks in patients with CRC by retrieving and analysis of RNA-seq data from The Cancer Genome Atlas (TCGA). Furthermore, we proposed numerous potential diagnostics, and prognostic lncRNA biomarkers such as *SLC16A1-AS1*, *AC087388.1*, and *ELFN1-AS1* which indicated promising results. In the present study, we investigated these candidate lncRNAs in our patients, and finally, we demonstrated the tumorigenic role of lncRNA *AC087388.1* in CRC cells.

## Methods

### Patients and tissue samples

The CRC tissues were collected by non-random sampling at the Reza radiotherapy, and oncology center, Mashhad, Iran. The age of the patients ranged from 24 to 83 years (mean age 57.25). A total of 40 CRC adenocarcinoma tissue samples were collected and confirmed by the pathological department. Informed consent was completed by participants at the beginning of the project. The study was approved by the Ethical Committee of Mashhad University of Medical Sciences (Code: IR.MUMS.MEDICAL.REC.1399.156).

### Cell culture

The human SW-48 and HT-29 CRC cell lines were obtained from the National Cell Bank of Iran (NCI, Tehran, Iran). The cells were cultured, according to ATCC (the American Type Culture Collection) recommendations, in RPMI-1640 medium (for SW48), and DMEM (For HT29) medium, both media from Cegrogen Biothech GmbH, Germany supplemented with 10% fetal bovine serum (FBS, Biosera, France) and 1% penicillin–streptomycin antibiotics (Biosera, France) in a humidified incubator in 5% CO2 at 37 °C. The cells were regularly checked for mycoplasma contamination.

### Quantitative Real-time PCR

RNA extraction was conducted by AccuZol™ (Bioneer, Korea) from the tissues and the cell lines. The quality and quantity of RNA extraction were evaluated by the 2% gel electrophoresis and a Nanodrop (Thermo Scientific, USA), respectively. cDNA synthesis was performed by the AccuPower RocketScript™ kit (Bioneer, Korea) according to the manual instruction. The total volume for this reaction was 20 μl that included 1 μg of total RNA. Quantitative Real-time PCR was applied to assess the RNA expression in the cells and tissues by a LightCycler® 96 System (Roche Life Science, Germany) using SYBR green-based kit, RealQ Plus Master Mix Green (Ampliqon, Copenhagen, Denmark). The total volume was 20 μL, including 10 μL of SYBR Green, 1 μL of primer (5 pmol), 2 μL of cDNA, and DEPC water. Thermal cycling conditions were comprised of an activation step at 95 °C for 15 min, followed by 40 cycles, including a denaturation step at 95 °C for 10 s and at 58 °C and 60 °C for 30 s for annealing and extension, respectively. The primer sequences of the target genes are listed in Table S1. *GAPDH* gene expression was considered as the reference gene. For calculation of relative expression, the 2^–ΔΔCT^ formula was used.

### Cell transfection

*AC087388.1* small hairpin RNA (shRNA) was synthesized by Metabion (Munich, Germany). The sequence was 5′-GCAAGAATGAGTATATCTATACCTGACCCATATAGATATACTCATTCTTGCTTTTT -3′. A scrambled negative control shRNA was also ordered from Metabion (Munich, Germany). The sequence was 5′- CCGGTACCTCACGTCAGTGGTGATATAGATCAAGAGTCTATATCACCACTGACGTTTTG -3′. The cells lines were incubated with either *AC087388.1* shRNA (shRNA) or negative control shRNA (as control) using polyethylenimine (PEI) transfection reagent (Merck KGaA, Darmstadt, Germany) according to the manufacturer protocol.

### Cell viability assay

The CRC cells were cultures into 96 well plates (1 × 10^4^ cells/well) for 24 h, 48 h, 72 h, 96 h, and 120 h. Following, the percentage of viable cells was determined by MTT (3-[4,5-dimethylthiazol-2-yl]-2,5 diphenyl tetrazolium bromide) assay, as 10 μl of MTT solution (5 mg/ml; Sigma) was added to each well and incubated with 5% CO2 at 37 °C for 4 h. Then, the supernatant was removed, and 100 μl of DMSO was added to each well as a solvent. Cell viability percentage was assessed by spectrophotometry at 570 and 630 nm using an absorbance microplate reader (BioTek ELx800, USA).

### Apoptosis assay

Annexin V and PI staining was carried out using Annexin V/PI-FITC apoptosis detection kit (MabTaq, Germany) according to the manufacturer’s protocol. The results were analyzed using a Partec PAS III flow cytometer (Partec) and WindowsTM FloMax® software (Partec).

### Colony formation assay

For colony formation assay, the CRC single-cell suspensions were cultured in 6-well collagen-coated plates (100 cells/well). The plates were further incubated for 7 days, and colonies were stained with 0.5% crystal violet and counted under an inverted microscope.

### Wound healing assay

Approximately 1 × 10^4^ the CRC cells were seeded into six-well collagen-coated plates. After overnight incubation, a linear wound was made in the confluent monolayer with a pipette tip. The cultures were washed with 1X phosphate buffer saline (PBS). The migration area was scanned after 5 days by an inverted microscope.

### Invasion assay

For evaluating the invasion ability of the cancer cells, transwell culture system were carried out. The CRC cell suspension was seeded (1 × 10^5^ cells/well) with a serum-free medium and cultured in the upper chamber of transwell cell culture chambers (8 mm pore size, Corning Inc., USA) precoated with Matrigel (BD Biosciences, USA). However, the lower chamber was filled with the medium containing 10% FBS. After 48 h incubation, the non-invasive cells remaining in the upper chamber were removed using a cotton swab and cells which passed through the inserts in the lower chamber were fixed with methanol and stained with 5% of crystal violet staining solution at room temperature for 20 min. A camera-equipped light microscope (Olympus, Japan) was applied counting the cells in the lower chamber. The number of invasive tumor cells was counted from five randomly selected 20 × fields per chamber for each assay which was conducted in triplicate.

### *In silico* functional enrichment analysis

For more illustration, the functional enrichment analysis of lncRNA *AC087388.1* was carried out by applying an online tool, ENCORI: The Encyclopedia of RNA Interactomes (http://starbase.sysu.edu.cn/) to demonstrate considerable CE-RNA networks and KEGG (Kyoto Encyclopedia of Genes and Genomes) signaling pathway analysis [22].

### Statistical analysis

All data are presented as mean ± standard deviation (SD) and were evaluated in triplicate against control and collected from three independent experiments. Data were graphed and analyzed by GraphPad Prism Software 7.0 using a two-tailed Student’s t-test for comparing the means between two independent groups, respectively. ROC curve analysis was conducted by SPSS v21. ROC curve was calculated according to the sample of the patients and counterpart control group, and the events was considered as tumor positive participants. *P-value* < 0.05 was considered as a statistically significant threshold.

## Results

In our previous study [4], we retrieved the public RNA-seq, miR-seq, and corresponding clinical data of 459 patients with CRC (primary tumor: 459, and adjacent normal solid tissue: 41) from the TCGA database. The differential gene expression was conducted by the “limma” package in R. Briefly, we demonstrated that 2995 mRNAs, 205 lncRNAs, and 345 miRNAs were differentially expressed in CRC. Gene ontology (GO) and KEGG signaling pathway were conducted and we demonstrated that the main number of the differentially expressed genes were enriched in important pathways in CRC. Furthermore, protein–protein interaction (PPI) was constructed by the STRING database, indicating that the *CDKN2A*, *CCND1*, *MYC*, *E2F*, *CDK4*, *BRCA2*, *CDC25B*, and *CDKN1A* proteins were the imperative signaling hubs. In addition, ceRNA network data showed the lncRNA-miRNA-mRNA interaction in the CRC patients (Tables S2 & S3). The diagnostic and prognostic values were evaluated for differentially expressed genes and finally, the data suggested 14 lncRNA as potential novel biomarkers in CRC. The data were sorted according to diagnostic and prognostic values, and the top three genes (lncRNA *SLC16A1-AS1* (chr1:112,956,415–112,964,072, intergenic), *AC087388.1* (chr17:7,685,260–7,686,371, intronic), *ELFN1-AS1* (chr7:1,738,630–1,742,310, intronic) were selected for further investigation in the current study. We investigated the lncRNAs expression in collected CRC samples and determined the role of lncRNA *AC087388.1* in CRC tumorigenesis.

### Forty patients were enrolled in the study

Forty CRC patients were encompassed in this study. All the patients’ tumors were CRC adenocarcinoma (with different grades). Twenty-three patients were male and 17 of them were female. According to the median age of the patients, 20 ones were more than 58 years and 20 were equal or less than 58 years old. Other features of the patients including tumor size, TNM staging, grading, *KRAS*, *BRAF*, and *NRAS* mutation status were summarized in Table 1.

**Table 1:** Clinicopathological characteristics of CRC patients

Characteristics	N	%
**# Case**	40	100
**SEX**		
Male	23	57.5
Female	17	42.5
**Age (y)**		
58 >	20	50
≤ 58	20	50
**Tumor size (cm)**		
4.5 >	18	45
≤ 4.5	22	55
**TNM**		
I	6	15
IIA	7	17.5
IIB	2	5
IIC	1	2.5
IIIA	2	5
IIIB	11	27.5
IIIC	10	25
NA	1	2.5
**Adenocarcinoma Grading**		
I	15	37.5
II	19	47.5
III	6	15
***KRAS mutation***		
positive	19	47.5
Negative	21	52.5
***BRAF mutation***		
positive	9	22.5
Negative	31	77.5
***NRAS mutation***		
positive	1	2.5
Negative	39	97.5

### LncRNA *SLC16A1-AS1, AC087388.1, and ELFN1-AS1* showed overexpression in the CRC tissues

**Fig. 1:**
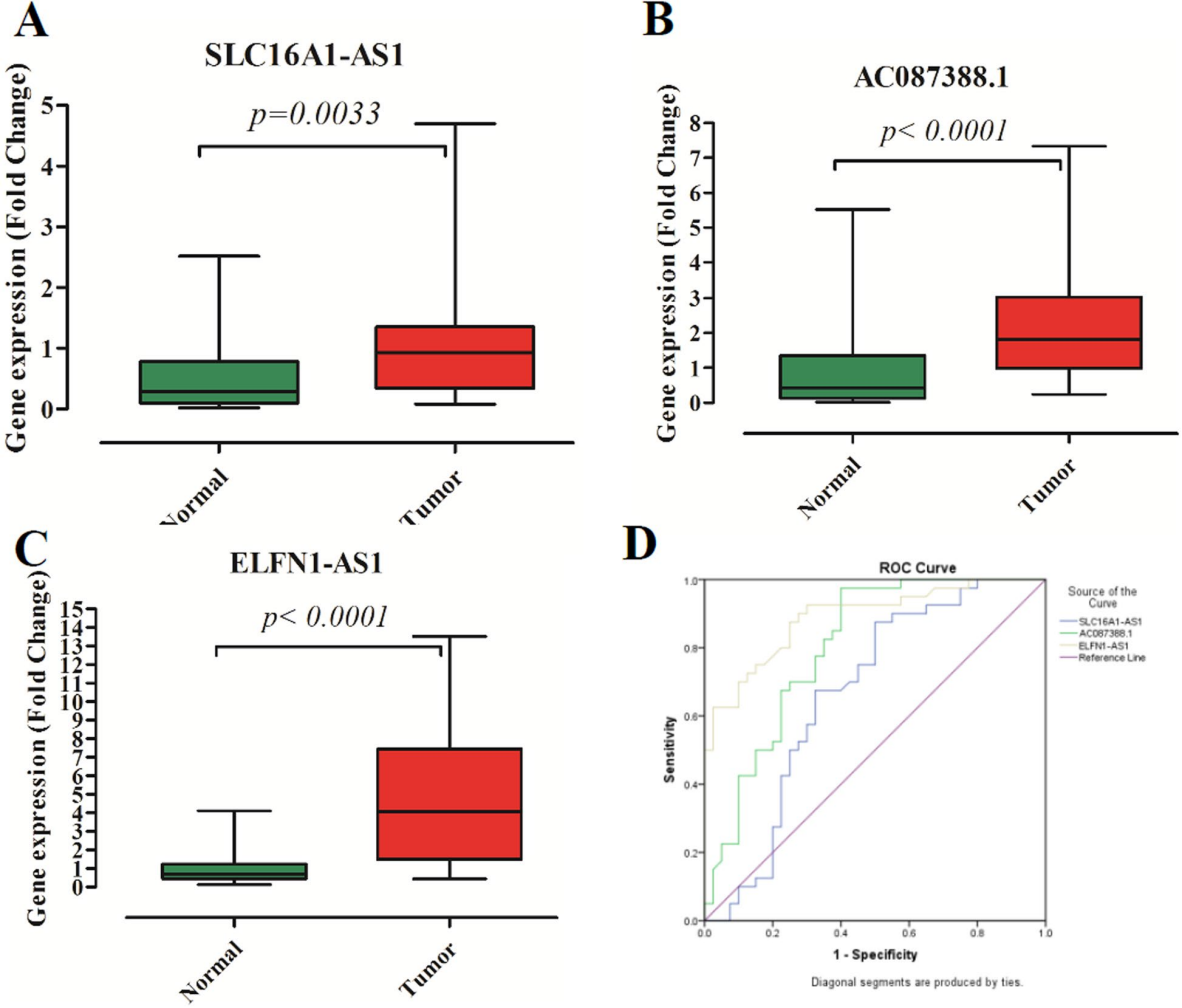
The expression of LncRNA *SLC16A1-AS1*, *AC087388.1*, and *ELFN1-AS1* in the patients. **A** The expression of lncRNA *SLC16A1-AS1.* **B** The expression of lncRNA *AC087388.1.* **C** The expression of lncRNA *ELFN1-AS1.* **D** ROC curves analysis of the lncRNAs

To explore the lncRNA *SLC16A1-AS1, AC087388.1*, and *ELFN1-AS1* expressions in the CRC patients, Quantitative Real-time PCR was applied. The results demonstrated that the lncRNAs were significantly overexpressed in the CRC tissues in comparison to their normal counterpart margins (Fig. 1). Furthermore, we compared the TCGA lncRNA expression data to our patients. They indicated a similar pattern in the same direction (Table S4). Moreover, for determining diagnostic values, ROC curve analyses were conducted. All lncRNAs had significant Area Under Curve (AUC) values. The data are presented in Fig. 1 and Table 2.

**Table 2:** ROC curve analysis of the lncRNAs

LncRNA	AUC	Std. Error	*p*-value	95% Confidence Interval	
				**Lower Bound**	**Upper Bound**
***SLC16A1-AS1***	0.668	0.063	0.01	0.545	0.791
***AC087388.1***	0.802	0.05	0	0.704	0.9
***ELFN1-AS1***	0.889	0.036	0	0.818	0.96

Furthermore, we evaluated the gene expression in the different groups of patients according to clinicopathological characteristics. The data showed that the expression of lncRNA *AC087388.1* in age 58 < is higher than ≤ 58 years, and expression of lncRNA *SLC16A1-AS1* in *BRAF* negative mutation is significantly higher than *BRAF* positive group. All the data are presented in Table 3. Moreover, we assessed the association of the lncRNAs expression and clinicopathological characteristics. The lncRNAs expression were divided into low and high expressions according to median expression. The data demonstrated that an increase in age was associated with a significantly high expression of the lncRNA *AC087388.1*. However, the results did not demonstrate any significant association between the high or low expression and clinicopathological characteristics in the patients. The data are summarized in Table 4.

**Table 3:** The gene expression according to clinicopathological characteristics

	***SLC16A1-AS1***		*P*-value	***AC087388.1***		*P*-value	***ELFN1-AS1***		*P*-value
	**Mean**	**Std. Error**		**Mean**	**Std. Error**		**Mean**	**Std. Error**	
**Sex**									
Male	0.89	0.32	0.14	2.33	0.20	0.97	4.29	0.31	0.22
Female	1.39	0.32		2.51	0.32		5.23	0.24	
**Age (y)**									
58 <	0.97	0.34	0.37	2.82	0.24	**0.02**	4.59	0.30	0.94
≤ 58	1.25	0.32		1.98	0.25		4.82	0.30	
**Tumor size (cm)**									
4.5 >	0.97	0.34	0.36	2.63	0.30	0.63	4.59	0.33	0.95
≤ 4.5	1.23	0.32		2.22	0.21		4.80	0.27	
**TNM**									
I	0.62	0.69	0.25	2.87	0.65	0.87	4.36	0.61	0.88
II	1.78	0.56		1.92	0.34		5.26	0.54	
III	0.94	0.25		2.50	0.21		4.55	0.23	
**Grade**									
I	1.21	0.46	0.79	2.28	0.32	0.68	4.56	0.39	0.51
II	1.11	0.33		2.34	0.23		5.26	0.27	
III	0.85	0.28		2.96	0.50		3.36	0.49	
***KRAS* mutation**									
positive	1.17	0.33	0.25	2.60	0.22	0.56	5.26	0.28	0.57
Negative	1.05	0.34		2.25	0.30		4.22	0.32	
***BRAF* mutation**									
positive	0.46	0.24	**0.01**	2.35	0.21	0.96	4.06	0.25	0.69
Negative	1.3	0.47		2.43	0.36		4.90	0.35	
***NRAS* mutaion**									
positive		0.24	0.97		0.18	0.36		0.21	0.46
Negative	1.11			2.39			4.77

**Table 4:** Association of the gene expression and demographic data

**Characteristics**	***SLC16A1-AS1***				***AC087388.1***				***ELFN1-AS1***			
	**Low**	**High**	**OR**	***P*-value**	**Low**	**High**	**OR**	***P*-value**	**Low**	**High**	**OR**	***P*-value**
**Sex**												
Male	13	10	0.538	0.337	12	11	0.815	0.749	13	10	0.5	0.337
Female	7	10			8	9			7	10		
**Age (y)**												
58 <	12	8	0.444	0.206	6	14	**5.444**	**0.011**	10	10	1	1
≤ 58	8	12			14	6			10	10		
**Tumor size (cm)**												
4.5 >	12	6	0.286	0.057	8	10	1.5	0.535	12	10	1.5	0.525
≤ 4.5	8	14			12	10			8	10		
**TNM**												
I + II	8	8	0.917	0.894	9	7	1.671	0.433	7	9	0.7	0.605
III	12	11			10	13			12	11		
**Grade**												
I	9	6	2.571	0.197	8	7	1.029	0.968	7	8	1	0.968
II	7	12	0.292	0.199	10	9	2.222	0.409	9	10	0.5	0.409
III	4	2	0.75	0.776	2	4	2.286	0.407	4	2	0.4	0.407
***KRAS* mutation**												
positive	8	11	1.833	0.342	9	10	1.222	0.752	10	9	0.8	0.752
Negative	12	9			11	10			10	11		
***BRAF* mutation**												
positive	7	2	0.206	0.058	5	4	0.75	0.705	5	4	0.8	0.705
Negative	13	18			15	16			15	16		
***NRAS* mutation**												
positive	1	0	0.487	0.311	1	0	0.487	0.311	1	0	0.5	0.311
Negative	19	20			19	20			19	20		

### *AC087388.1* small hairpin RNA (shRNA) downregulated lncRNA *AC087388.1* in CRC cells

**Fig. 2:**
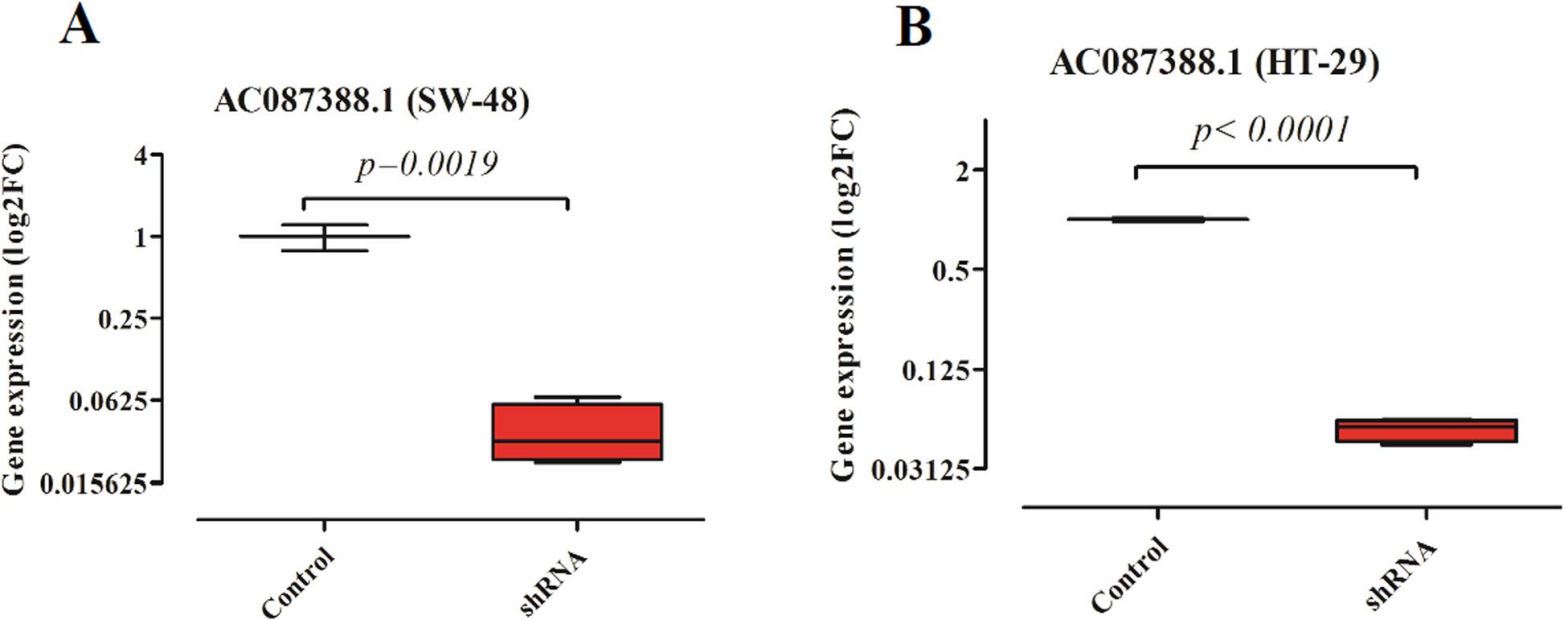
Downregulation of lncRNA *AC087388.1* in CRC cells. **A** SW-48. **B** HT29

In the next step, by considering the top list lncRNAs and a lack of sufficient studies on the novel candidate lncRNAs, we selected lncRNA *AC087388.1* for further investigation. By applying shRNA against *AC087388.1* in the CRC cell lines (SW-48 and HT-29), we established stable cell lines producing the shRNA constantly (shRNA). In this study, we used a scrambled shRNA as a negative control (Control). The data illustrated that the shRNA significantly reduced the expression of the lncRNA *AC087388.1* in comparison to the control in both cell lines. Figure 2 presents the data.

### Downregulation of lncRNA *AC087388.1* suppresses cell proliferation and viability

**Fig. 3:**
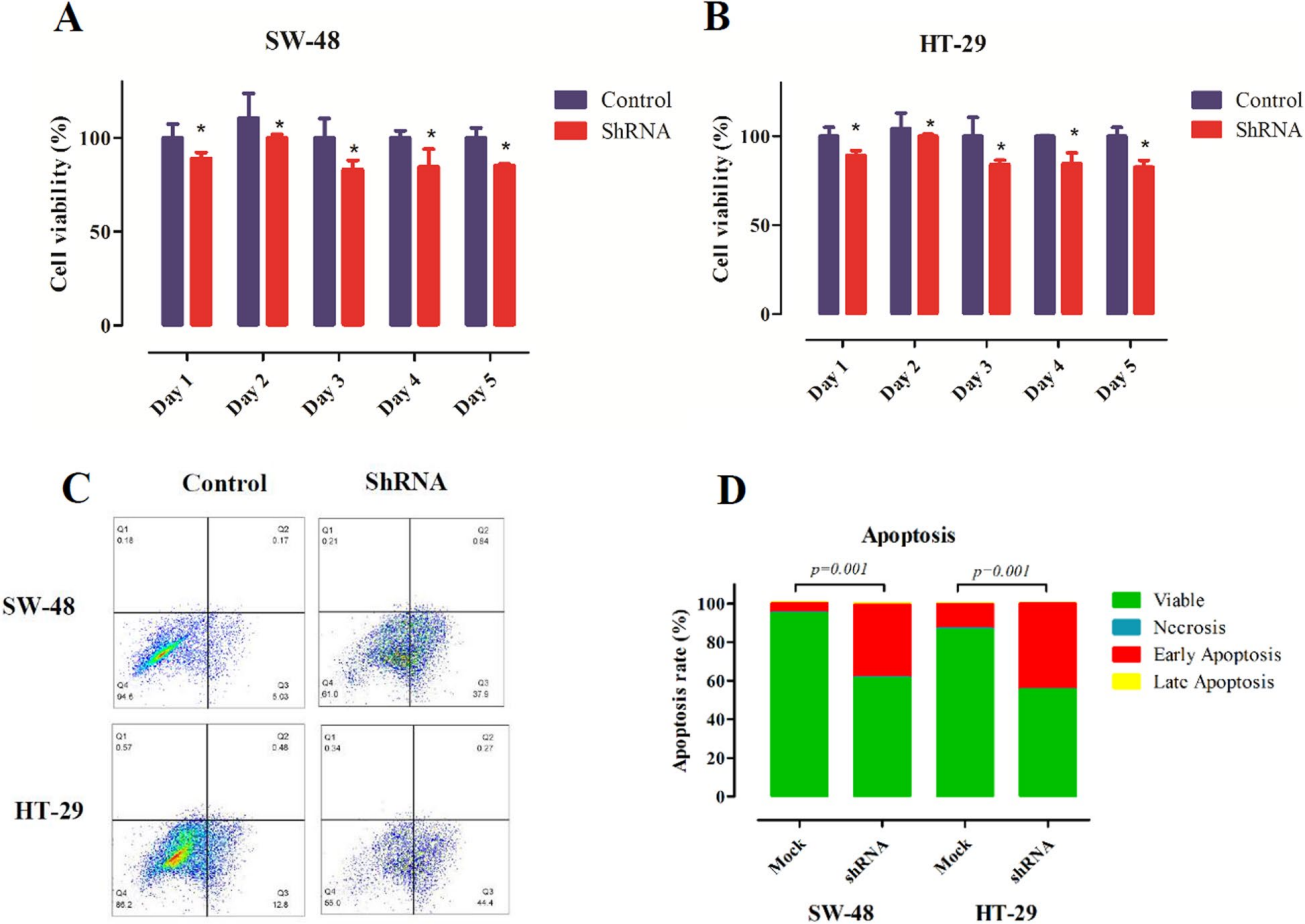
Downregulation of lncRNA *AC087388.1* remarkably decreased cell viability. **A** MTT assay for SW-48 cell. **B** MTT assay for HT-29 cell. **C** Apoptosis fraction graph of the cells, apoptotic cell death was measured by annexin V staining after 24 h. Annexin V-positive cells are considered early apoptotic, whereas PI uptake indicates necrosis. Cells positive for both stains are considered apoptotic cells. **D** The percentage of the viable, necrosis, early, and late apoptosis

To evaluate the cell proliferation and viability in downregulation condition of lncRNA *AC087388.1*, we applied MTT and apoptosis assays. According to the MTT assay results, the proliferation of the shRNA-treated cells was significantly suppressed in comparison to the controls. Also, the apoptosis data showed that downregulation of lncRNA *AC087388.1* remarkably decreased cell viability and increased early apoptosis in comparison to the control in the CRC cells. The data are presented in Fig. 3.

### Downregulation of lncRNA *AC087388.1* suppresses cellular growth and colony formation capability

**Fig. 4:**
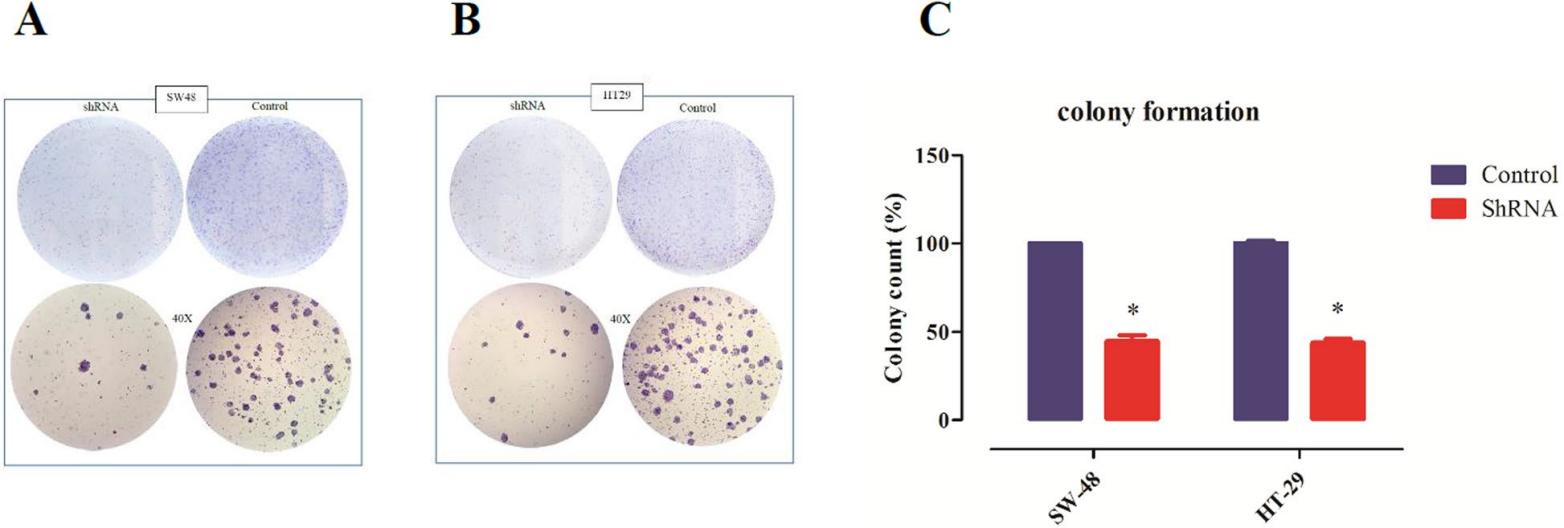
Downregulation of lncRNA *AC087388.1* suppressed cell growth and colony formation capability. **A** SW-48 cell line. **B **HT-29 cell line. **C** The colony counts parentage of the cells

Cell growth and colony formation capability of single-cell suspension were assessed by colony formation assay. The data demonstrated that downregulation of lncRNA *AC087388.1* significantly suppressed cell growth and colony formation capability in comparison to the control. The data are presented in Fig. 4.

### Downregulation of lncRNA *AC087388.1* attenuates cell motility and invasion

**Fig. 5:**
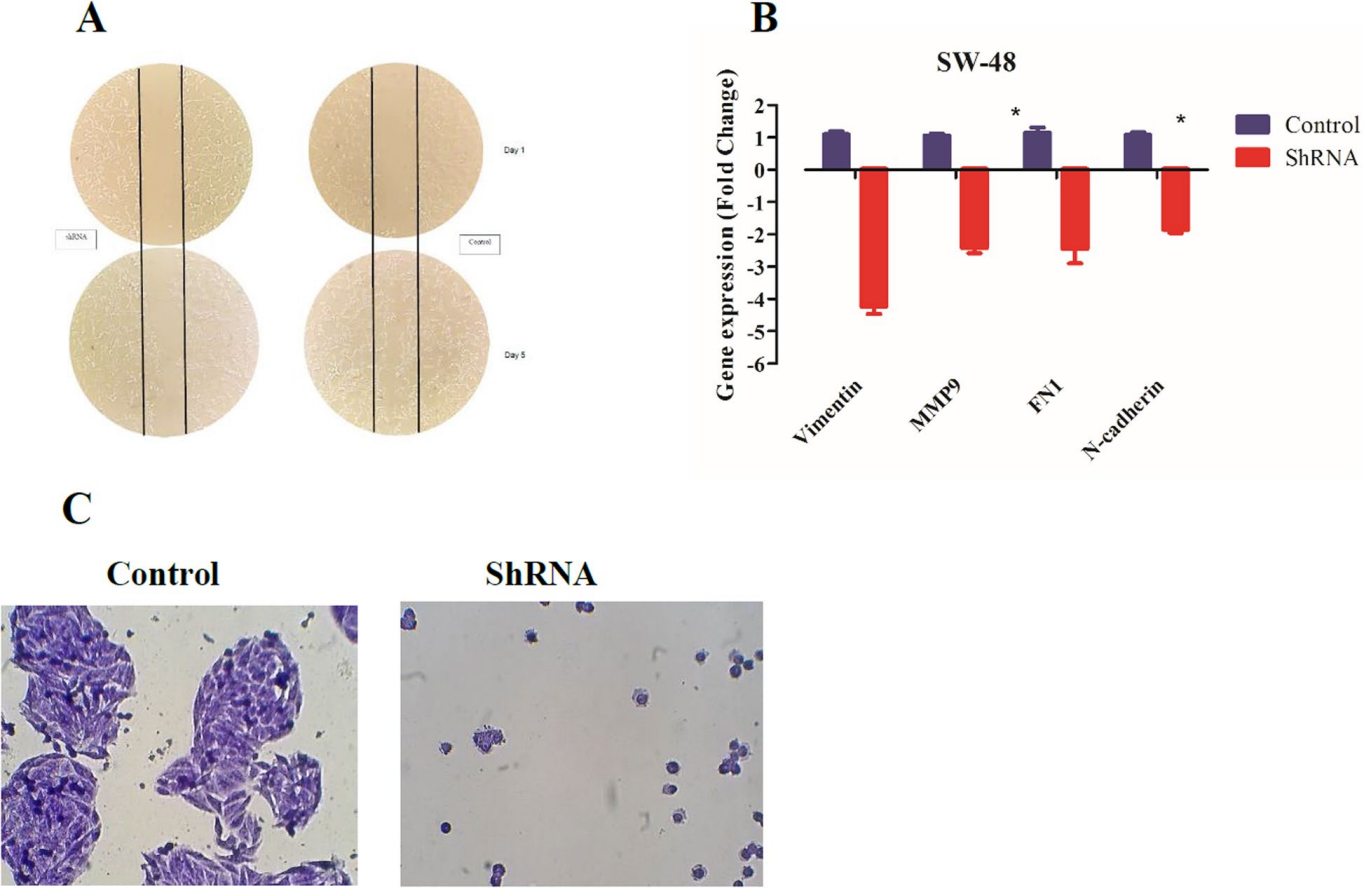
Downregulation of lncRNA *AC087388.1* attenuates cell motility and invasion ability. **A** Downregulation of lncRNA *AC087388.1* inhibited the motility of the SW-48 cells as demonstrated by reduced width in would healing assay. **B** The expression of vimentin, MMP9, FN1, and N-Cadherin were significantly reduced in the downregulation of lncRNA *AC087388.1* condition. **C** As the transwell cell migration and invasion assay represent, the downregulation of lncRNA *AC087388.1* inhibits invasion and migration of the CRC cells

We investigated the motility and migration ability of the CRC shRNA-treated cells. Wound healing assay revealed that the downregulation of lncRNA *AC087388.1* attenuated motility of the SW-48 cells in comparison with the control group (Fig. 5A). In addition, expression of migration and invasion contributor genes were evaluated by Quantitative Real-time PCR. The data demonstrated that the downregulation of lncRNA *AC087388.1* remarkably decreased expression of *Vimentin, MMP9, FN1,* and *N-Cadherin* in the SW-48 cell line (Fig. 5B). Furthermore, hereby in transwell cell migration and invasion assay, we showed that the cell invasion and migration of the SW-48 cells decreased (Fig. 5C).

### LncRNA *AC087388.1* has a contribution in canonical signaling pathways in cancer

**Fig. 6:**
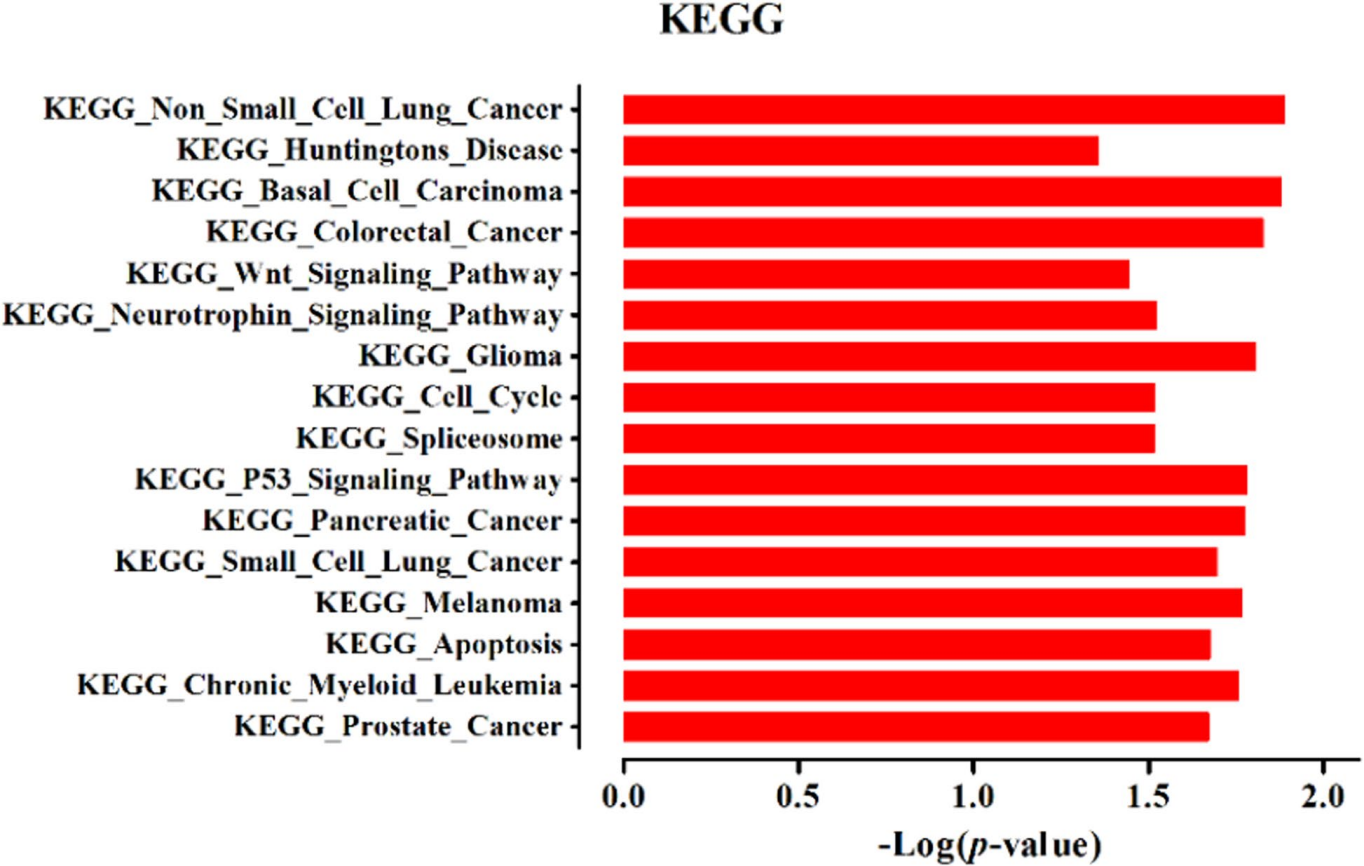
KEGG signaling pathway analysis of the lncRNA *AC087388.1* target genes

For further investigation on lncRNA *AC087388.1* roles in CRC and to demonstrate the downstream signaling pathways, we conducted *In-silico* functional studies. *In-silico* functional enrichment analysis of the lncRNA *AC087388.1* by ENCORI online tool, demonstrated that the lncRNA *AC087388.1* can regulate varieties of the genes in the human cells (Table 5). Furthermore, the gene set enrichment by KEGG pathway analysis showed that many of the genes were enriched in crucial signaling pathways in cancer such as the p53 signaling pathway, Wnt Signaling pathway, apoptosis, and cell cycle. The data are presented in Fig. 6.

**Table 5:** LncRNA *AC087388.1* potential targets in CE-network (top 50 term are presented)

ceRNAid	ceRNAname	ceRNAgeneType	*p*-value	FDR
ENSG00000106077	ABHD11	protein_coding	7.80E-07	1.06E-04
ENSG00000113262	GRM6	protein_coding	8.50E-07	1.06E-04
ENSG00000152223	EPG5	protein_coding	1.77E-06	1.06E-04
ENSG00000279466	AC073911.2	TEC	1.85E-06	1.06E-04
ENSG00000068489	PRR11	protein_coding	2.82E-06	1.06E-04
ENSG00000100209	HSCB	protein_coding	6.95E-06	1.58E-04
ENSG00000196705	ZNF431	protein_coding	1.19E-05	2.32E-04
ENSG00000223502	AL731537.1	antisense	1.26E-05	2.32E-04
ENSG00000215014	AL645728.1	lincRNA	1.26E-05	2.32E-04
ENSG00000152931	PART1	lincRNA	1.27E-05	2.32E-04
ENSG00000259488	AC023355.1	antisense	1.29E-05	2.32E-04
ENSG00000130921	C12orf65	protein_coding	1.58E-05	2.32E-04
ENSG00000133997	MED6	protein_coding	1.58E-05	2.32E-04
ENSG00000171490	RSL1D1	protein_coding	1.62E-05	2.32E-04
ENSG00000243410	PSMD6-AS1	antisense	1.76E-05	2.32E-04
ENSG00000277511	AC116407.2	lincRNA	1.76E-05	2.32E-04
ENSG00000169288	MRPL1	protein_coding	1.87E-05	2.32E-04
ENSG00000181192	DHTKD1	protein_coding	2.10E-05	2.32E-04
ENSG00000243667	WDR92	protein_coding	2.21E-05	2.32E-04
ENSG00000226987	AL157938.1	processed_pseudogene	2.48E-05	2.32E-04
ENSG00000128534	LSM8	protein_coding	2.81E-05	2.32E-04
ENSG00000169684	CHRNA5	protein_coding	2.81E-05	2.32E-04
ENSG00000011275	RNF216	protein_coding	3.79E-05	2.32E-04
ENSG00000180979	LRRC57	protein_coding	4.15E-05	2.35E-04
ENSG00000110075	PPP6R3	protein_coding	4.29E-05	2.35E-04
ENSG00000111196	MAGOHB	protein_coding	4.30E-05	2.35E-04
ENSG00000280195	AC245140.2	antisense	4.39E-05	2.35E-04
ENSG00000223891	OSER1-AS1	lincRNA	4.39E-05	2.35E-04
ENSG00000181904	C5orf24	protein_coding	4.55E-05	2.35E-04
ENSG00000173011	TADA2B	protein_coding	4.63E-05	2.35E-04
ENSG00000040633	PHF23	protein_coding	5.18E-05	2.35E-04
ENSG00000198863	RUNDC1	protein_coding	5.40E-05	2.35E-04
ENSG00000116254	CHD5	protein_coding	6.04E-05	2.49E-04
ENSG00000137831	UACA	protein_coding	6.51E-05	2.60E-04
ENSG00000277692	AL121583.1	lincRNA	6.91E-05	2.69E-04
ENSG00000233693	AL357568.1	antisense	6.91E-05	2.69E-04
ENSG00000265139	AC005899.3	lincRNA	6.91E-05	2.69E-04
ENSG00000040487	PQLC2	protein_coding	7.01E-05	2.69E-04
ENSG00000269588	AC011500.2	unprocessed_pseudogene	7.20E-05	2.69E-04
ENSG00000229676	ZNF492	protein_coding	7.84E-05	2.69E-04
ENSG00000118620	ZNF430	protein_coding	7.91E-05	2.69E-04
ENSG00000105708	ZNF14	protein_coding	8.87E-05	2.87E-04
ENSG00000188227	ZNF793	protein_coding	8.94E-05	2.87E-04
ENSG00000103449	SALL1	protein_coding	1.11E-04	3.43E-04
ENSG00000235931	LINC01553	lincRNA	1.11E-04	3.43E-04
ENSG00000144713	RPL32	protein_coding	1.24E-04	3.67E-04
ENSG00000146072	TNFRSF21	protein_coding	1.27E-04	3.67E-04
ENSG00000163625	WDFY3	protein_coding	1.27E-04	3.67E-04
ENSG00000062370	ZNF112	protein_coding	1.38E-04	3.83E-04
ENSG00000134375	TIMM17A	protein_coding	1.54E-04	4.18E-04

## Discussion

CRC is one of the common leading cancer-related deaths with an increasing trend in the world [23]. The early-stage diagnosis of CRC can provide the desired outcome in the patients [24]. Despite huge efforts in developing diagnostic and prognostic methods, a large body of patients is diagnosed in advanced stages, which have shown frustrating outcomes [7]. Thus, an in-depth understanding of CRC’s underlying mechanisms is pivotal. Recently, a number of the investigation highlighted the roles and function of lncRNAs in various cancers particularly in CRC [4, 6, 15, 25, 26]. Recently, we demonstrated overexpression of lncRNA *SLC16A1-AS1*, *AC087388.1*, and *ELFN1-AS1* in CRC patients on the report of the TCGA public database. Furthermore, due to desired prognostic and diagnostic outcome, we indicated that lncRNA *SLC16A1-AS1*, *AC087388.1*, and *ELFN1-AS1* could be considered as potential biomarkers in CRC patients [4]. In the current study, we broadly explored the role and the in vitro function of the lncRNA *AC087388.1* in CRC cells. The data showed overexpression of lncRNA *AC087388.1* in our CRC patients. Furthermore, the data demonstrated that downregulation of lncRNA *AC087388.1* inhibits cell proliferation, growth, and invasion in CRC cells.

Cell proliferation and growth are important in tumorigenesis, being the hallmark of CRC [27, 28]. A variety of signaling pathways such as phosphatidylinositol-3-kinase/protein kinase B (*PI3K*/*AKT*) play key roles in cancer cell growth and proliferation [29, 30]. A large body of investigation has shown that lncRNA could regulate cell proliferation and growth in CRC. For instance, it has been shown that the novel lncRNA *LINC00460* has been associated with large tumor size, advanced stages of cancer, and poor prognosis in the CRC patients, and has an impact on cell proliferation and apoptosis via sponging *EZH2* and *miR-149-5p* to upregulating *KLF2* and *CUL4A* in CRC, respectively [31]. Furthermore, lncRNA *CRNDE (Colorectal Neoplasia Differentially Expressed)* has been illustrated overexpression in CRC patients and has been associated with worse clinicopathological outcomes and poor prognosis. lncRNA *CRNDE* enhances tumorigenesis through epigenetically silencing *dual-specificity phosphatase 5* (*DUSP5*) and *CDKN1A* by recruiting *EZH2* (*enhancer of zeste homolog 2*) in CRC cells [32]. Constant with previous studies, we presented that lncRNA *AC087388.1* overexpressed in our CRC patients. Moreover, it has been shown that the downregulation of lncRNA *AC087388.1* remarkably decreased cell proliferation, growth, and cell viability in CRC cells.

Another, crucial hallmark of cancer is invasion and metastasis [27]. Several investigations indicating different sorts of signaling pathways that have a main contribution to invasion and metastasis including epithelial NOTCH, MAPK, STAT3 signaling pathways [33–36]. Many studies have indicated the lncRNA regulatory effects on invasion and metastasis in CRC cells [37–39]. LncRNAs can control cell invasion and metastasis by regulating different signaling pathways such as PI3K/AKT signaling pathway, EGFR/MAPK pathway, and hypoxia-induced signaling pathway in CRC [40–42]. For instance, it has been demonstrated that lncRNA *SNHG5* enhances cell proliferation and metastasis by increasing *CREB5* through downregulating *miR-132-3p* in CRC cells [43]. According to our results, downregulating of lncRNA *AC087388.1* could attenuate cell mobility and invasion in the CRC cells. Furthermore, it reduced the colony formation ability of the cells from single CRC suspension cells. Tumor invasion and migration occur when the basement membranes and extracellular matrix (ECM) are dissolved by matrix metalloproteinases (MMPs) [44, 45]. MMPs are a group of zinc-dependent endopeptidases that work towards ECM turnover [46]. *Vimentin, MMP9, FN1,* and *N-Cadherin* are the well-known genes that have the main contribution to metastasis and EMT (Epithelial-to-mesenchymal transition) in cancer [47]. In this study, we showed that downregulation of *AC087388.1* remarkably reduced expression of invasion and migration genes including *Vimentin, MMP9, FN1,* and *N-Cadherin* in CRC cells which explained the invasive role of this lncRNA.

For more illustration of lncRNA *AC087388.1* roles in CRC *In-silico* functional study was applied to demonstrating the downstream signaling pathways and canonical signaling hubs. The *in-silico* analysis of our study noticeably demonstrated that the lncRNA *AC087388.1* could drive tumorigenesis in various cancers such as prostate cancer, chronic myeloid leukemia, melanoma, and CRC. There are varieties of crucial signaling pathways in CRC which have a major contribution to tumorigenesis such as the Wnt signaling pathway, neurotrophin signaling pathway, p53 signaling pathway [48–52]. In the present study, we reported that the lncRNA *AC087388.1* can control a variety of signaling pathways such as the Wnt signaling pathway, neurotrophin signaling pathway, cell cycle and apoptosis, and p53 signaling pathway.

## Conclusions

To the best of our knowledge, for the first time, we showed that lncRNA *AC087388.1* has an oncogenic role in tumorigenesis of CRC. lncRNA *AC087388.1* can be considered as a novel diagnostic and prognostic biomarker in CRC. This study sheds light for further investigation and paves the way for researchers in the field of cancer and lncRNA. Further investigations are needed to illustrate the detailed role of lncRNA *AC087388.1* in tumorigenesis particularly in CRC.

## Supplementary Information

**Additional file 1: Table S1.** The primer sequence sets of the genes.

**Additional file 2: Table S2. **The number of miRNA interactions to lncRNAs and mRNAs.

**Additional file 3: Table S3. **The miRNA targets to lncRNAs and mRNAs.

**Additional file 4: Table S4. **Comparisonof the TCGA gene expression with our patients.

## Data Availability

The datasets generated and/or analysed during the current study are available in the TCGA database repository, [https://portal.gdc.cancer.gov/].

## Abbreviations

lncRNA: Long non-coding RNA
CRC: Colorectal cancer
ATCC: The American Type Culture Collection
AUC: Area Under Curve
GI: Gastrointestinal
gFOBT: Guaiac fecal occult blood test
FIT: Fecal immunochemical test
TCGA: The Cancer Genome Atlas
NCBI: National Cell Bank of Iran
ATCC: The American Type Culture Collection
FBS: Fetal bovine serum
PEI: Polyethylenimine
MTT: 3-[4,5-Dimethylthiazol-2-yl]-2,5 diphenyl tetrazolium bromide
PBS: Phosphate buffer saline
ENCORI: The Encyclopedia of RNA Interactomes
SD: Standard deviation
GO: Gene ontology
PPI: Protein–protein interaction
AUC: Area Under Curve
KEGG: Kyoto Encyclopedia of Genes and Genomes
*PI3K*/*AKT*: Phosphatidylinositol-3-kinase/protein kinase B
*CRNDE*: *Colorectal Neoplasia Differentially Expressed*
*DUSP5*: *dual-specificity phosphatase 5*
*EZH2*: *Enhancer of zeste homolog 2*
MMPs: Matrix metalloproteinases
EMT: Epithelial-to-mesenchymal transition
