# Supplementary material for: Long non-coding RNA AC087388.1 as a novel biomarker in colorectal cancer
Source: BMC Cancer. 2022 Feb 21;22:196. doi: 10.1186/s12885-022-09282-0 (PMC8862536; doi:10.1186/s12885-022-09282-0)
Supplement: Supplementary file 1 — Additional file 1: Table S1. The primer sequence sets of the genes. [file 12885_2022_9282_MOESM1_ESM.docx]

| **Primer** | **Forward** | **Reverse** |
| --- | --- | --- |
| SLC16A1-AS1 | GGGAGACTTAGGCACAAATTAACC | ATGTTGGTGTGCTTGAAATCTTCC |
| AC087388.1 | TGATGCCTTCCGGGTCAC | TGTTTTCATTCTATAGGAAGT |
| ELFN1-AS1 | AATAACCCAAAGTCAAGCTGAC | TTGGAGCAGCCACTTAGAC |
| FN1 | CGGTGGCTGTCAGTCAAAG | AAACCTCGGCTTCCTCCATAA |
| Vimentin | CGCCAGATGGGTGAAATGG | ACCAGAGGGAGTGAATCCACA |
| N-cadherin | ACAGTGGCCACCTACAAAGG | CCGAGATGGGGTTGATAATG |
| E-cadherin | CCCACCACGTACAAGGGTC | CTGGGGTATTGGGGGCATC |
| MMP-9 | TGTACCGCTATGGTTACACTCG | GGCAGGGACAGTTGCTTCT |
| GAPDH | CATGTTCGTCATGGGTGTGAAC | CACAGTCTTCTGGGTGGCAG |

**Table S1.** The primer sequence sets of the genes.
