# Supplementary material for: Long non-coding RNA AC087388.1 as a novel biomarker in colorectal cancer
Source: BMC Cancer. 2022 Feb 21;22:196. doi: 10.1186/s12885-022-09282-0 (PMC8862536; doi:10.1186/s12885-022-09282-0)
Supplement: Supplementary file 2 — Additional file 2: Table S2. The number of miRNA interactions to lncRNAs and mRNAs. [file 12885_2022_9282_MOESM2_ESM.docx]

Table S2. The number of miRNA interactions to lncRNAs and mRNAs

|  | Gene | Symbol | Type | Interaction no. |
| --- | --- | --- | --- | --- |
| 1 | ENSG00000025772 | TOMM34 | pc | 3 |
| 2 | ENSG00000047634 | SCML1 | pc | 4 |
| 3 | ENSG00000059588 | TARBP1 | pc | 4 |
| 4 | ENSG00000065320 | NTN1 | pc | 2 |
| 5 | ENSG00000065534 | MYLK | pc | 2 |
| 6 | ENSG00000066468 | FGFR2 | pc | 2 |
| 7 | ENSG00000066735 | KIF26A | pc | 3 |
| 8 | ENSG00000068650 | ATP11A | pc | 5 |
| 9 | ENSG00000070961 | ATP2B1 | pc | 5 |
| 10 | ENSG00000079335 | CDC14A | pc | 2 |
| 11 | ENSG00000082512 | TRAF5 | pc | 3 |
| 12 | ENSG00000097046 | CDC7 | pc | 4 |
| 13 | ENSG00000101955 | SRPX | pc | 3 |
| 14 | ENSG00000103034 | NDRG4 | pc | 2 |
| 15 | ENSG00000103257 | SLC7A5 | pc | 3 |
| 16 | ENSG00000105976 | MET | pc | 5 |
| 17 | ENSG00000106089 | STX1A | pc | 3 |
| 18 | ENSG00000106344 | RBM28 | pc | 1 |
| 19 | ENSG00000108821 | COL1A1 | pc | 5 |
| 20 | ENSG00000111913 | RIPOR2 | pc | 2 |
| 21 | ENSG00000111962 | UST | pc | 2 |
| 22 | ENSG00000113140 | SPARC | pc | 3 |
| 23 | ENSG00000114251 | WNT5A | pc | 2 |
| 24 | ENSG00000114270 | COL7A1 | pc | 3 |
| 25 | ENSG00000117385 | P3H1 | pc | 3 |
| 26 | ENSG00000119938 | PPP1R3C | pc | 2 |
| 27 | ENSG00000119969 | HELLS | pc | 3 |
| 28 | ENSG00000124225 | PMEPA1 | pc | 5 |
| 29 | ENSG00000126821 | SGPP1 | pc | 2 |
| 30 | ENSG00000129682 | FGF13 | pc | 2 |
| 31 | ENSG00000130600 | H19 | lnc | 12 |
| 32 | ENSG00000131389 | SLC6A6 | pc | 5 |
| 33 | ENSG00000134013 | LOXL2 | pc | 3 |
| 34 | ENSG00000134901 | KDELC1 | pc | 3 |
| 35 | ENSG00000134982 | APC | pc | 2 |
| 36 | ENSG00000135763 | URB2 | pc | 1 |
| 37 | ENSG00000136108 | CKAP2 | pc | 1 |
| 38 | ENSG00000136295 | TTYH3 | pc | 5 |
| 39 | ENSG00000137449 | CPEB2 | pc | 7 |
| 40 | ENSG00000137558 | PI15 | pc | 5 |
| 41 | ENSG00000137941 | TTLL7 | pc | 2 |
| 42 | ENSG00000138411 | HECW2 | pc | 5 |
| 43 | ENSG00000139163 | ETNK1 | pc | 5 |
| 44 | ENSG00000142632 | ARHGEF19 | pc | 3 |
| 45 | ENSG00000143515 | ATP8B2 | pc | 2 |
| 46 | ENSG00000143995 | MEIS1 | pc | 2 |
| 47 | ENSG00000146122 | DAAM2 | pc | 3 |
| 48 | ENSG00000148516 | ZEB1 | pc | 5 |
| 49 | ENSG00000152413 | HOMER1 | pc | 5 |
| 50 | ENSG00000155545 | MIER3 | pc | 7 |
| 51 | ENSG00000156218 | ADAMTSL3 | pc | 2 |
| 52 | ENSG00000157168 | NRG1 | pc | 3 |
| 53 | ENSG00000159167 | STC1 | pc | 5 |
| 54 | ENSG00000163235 | TGFA | pc | 2 |
| 55 | ENSG00000164176 | EDIL3 | pc | 2 |
| 56 | ENSG00000168077 | SCARA3 | pc | 2 |
| 57 | ENSG00000169851 | PCDH7 | pc | 2 |
| 58 | ENSG00000175592 | FOSL1 | pc | 4 |
| 59 | ENSG00000177732 | SOX12 | pc | 4 |
| 60 | ENSG00000179041 | RRS1 | pc | 1 |
| 61 | ENSG00000182983 | ZNF662 | pc | 3 |
| 62 | ENSG00000184371 | CSF1 | pc | 5 |
| 63 | ENSG00000196411 | EPHB4 | pc | 5 |
| 64 | ENSG00000197343 | ZNF655 | pc | 2 |
| 65 | ENSG00000198121 | LPAR1 | pc | 2 |
| 66 | ENSG00000198720 | ANKRD13B | pc | 6 |
| 67 | ENSG00000198910 | L1CAM | pc | 2 |
| 68 | ENSG00000204262 | COL5A2 | pc | 3 |
| 69 | ENSG00000211448 | DIO2 | pc | 3 |
| 70 | ENSG00000215417 | MIR17HG | lnc | 7 |
| 71 | ENSG00000220205 | VAMP2 | pc | 2 |
| 72 | ENSG00000234456 | MAGI2-AS3 | lnc | 2 |
| 73 | ENSG00000234741 | GAS5 | lnc | 2 |
| 74 | ENSG00000234912 | SNHG20 | lnc | 1 |
| 75 | ENSG00000244479 | OR2A1-AS1 | lnc | 1 |
| 76 | ENSG00000251562 | MALAT1 | lnc | 7 |
| 77 | ENSG00000255717 | SNHG1 | lnc | 3 |
| 78 | ENSG00000269821 | KCNQ1OT1 | lnc | 12 |
| 79 | hsa-miR-103a-3p | hsa-miR-103a-3p | mir | 3 |
| 80 | hsa-miR-107 | hsa-miR-107 | mir | 3 |
| 81 | hsa-miR-124-3p | hsa-miR-124-3p | mir | 2 |
| 82 | hsa-miR-1271-5p | hsa-miR-1271-5p | mir | 2 |
| 83 | hsa-miR-130a-3p | hsa-miR-130a-3p | mir | 20 |
| 84 | hsa-miR-130b-3p | hsa-miR-130b-3p | mir | 20 |
| 85 | hsa-miR-137 | hsa-miR-137 | mir | 2 |
| 86 | hsa-miR-138-5p | hsa-miR-138-5p | mir | 3 |
| 87 | hsa-miR-140-5p | hsa-miR-140-5p | mir | 2 |
| 88 | hsa-miR-148a-3p | hsa-miR-148a-3p | mir | 2 |
| 89 | hsa-miR-148b-3p | hsa-miR-148b-3p | mir | 2 |
| 90 | hsa-miR-152-3p | hsa-miR-152-3p | mir | 2 |
| 91 | hsa-miR-200b-3p | hsa-miR-200b-3p | mir | 3 |
| 92 | hsa-miR-200c-3p | hsa-miR-200c-3p | mir | 3 |
| 93 | hsa-miR-24-3p | hsa-miR-24-3p | mir | 3 |
| 94 | hsa-miR-29a-3p | hsa-miR-29a-3p | mir | 20 |
| 95 | hsa-miR-29b-3p | hsa-miR-29b-3p | mir | 20 |
| 96 | hsa-miR-29c-3p | hsa-miR-29c-3p | mir | 20 |
| 97 | hsa-miR-301a-3p | hsa-miR-301a-3p | mir | 8 |
| 98 | hsa-miR-301b-3p | hsa-miR-301b-3p | mir | 8 |
| 99 | hsa-miR-326 | hsa-miR-326 | mir | 4 |
| 100 | hsa-miR-330-5p | hsa-miR-330-5p | mir | 4 |
| 101 | hsa-miR-335-5p | hsa-miR-335-5p | mir | 2 |
| 102 | hsa-miR-3666 | hsa-miR-3666 | mir | 12 |
| 103 | hsa-miR-370-3p | hsa-miR-370-3p | mir | 2 |
| 104 | hsa-miR-374a-5p | hsa-miR-374a-5p | mir | 24 |
| 105 | hsa-miR-374b-5p | hsa-miR-374b-5p | mir | 24 |
| 106 | hsa-miR-421 | hsa-miR-421 | mir | 2 |
| 107 | hsa-miR-429 | hsa-miR-429 | mir | 3 |
| 108 | hsa-miR-4295 | hsa-miR-4295 | mir | 12 |
| 109 | hsa-miR-454-3p | hsa-miR-454-3p | mir | 19 |
| 110 | hsa-miR-485-5p | hsa-miR-485-5p | mir | 2 |
| 111 | hsa-miR-495-3p | hsa-miR-495-3p | mir | 2 |
| 112 | hsa-miR-506-3p | hsa-miR-506-3p | mir | 2 |
| 113 | hsa-miR-653-5p | hsa-miR-653-5p | mir | 2 |
| 114 | hsa-miR-7-5p | hsa-miR-7-5p | mir | 2 |
| 115 | hsa-miR-96-5p | hsa-miR-96-5p | mir | 2 |
