# Supplementary material for: Long non-coding RNA AC087388.1 as a novel biomarker in colorectal cancer
Source: BMC Cancer. 2022 Feb 21;22:196. doi: 10.1186/s12885-022-09282-0 (PMC8862536; doi:10.1186/s12885-022-09282-0)
Supplement: Supplementary file 3 — Additional file 3: Table S3. The miRNA targets to lncRNAs and mRNAs. [file 12885_2022_9282_MOESM3_ESM.docx]

Table S3. The miRNA targets to lncRNAs and mRNAs.

| From_Node | To_Node | Targets | Pearson - correlation | P-value |
| --- | --- | --- | --- | --- |
| ENSG00000255717 | hsa-miR-326 | SNHG1 | -0.71 | 0.000 |
| ENSG00000255717 | hsa-miR-330-5p | SNHG1 | -0.56 | 0.002 |
| ENSG00000255717 | hsa-miR-421 | SNHG1 | 0.75 | 0.000 |
| ENSG00000234456 | hsa-miR-374b-5p | MAGI2-AS3 | -0.54 | 0.004 |
| ENSG00000234456 | hsa-miR-374a-5p | MAGI2-AS3 | -0.51 | 0.009 |
| ENSG00000215417 | hsa-miR-4295 | MIR17HG | -0.76 | 0.000 |
| ENSG00000215417 | hsa-miR-130a-3p | MIR17HG | 0.54 | 0.006 |
| ENSG00000215417 | hsa-miR-454-3p | MIR17HG | 0.77 | 0.000 |
| ENSG00000215417 | hsa-miR-301a-3p | MIR17HG | 0.61 | 0.001 |
| ENSG00000215417 | hsa-miR-301b-3p | MIR17HG | 0.59 | 0.002 |
| ENSG00000215417 | hsa-miR-130b-3p | MIR17HG | 0.79 | 0.000 |
| ENSG00000215417 | hsa-miR-3666 | MIR17HG | -0.76 | 0.000 |
| ENSG00000234741 | hsa-miR-137 | GAS5 | -0.53 | 0.005 |
| ENSG00000234741 | hsa-miR-485-5p | GAS5 | -0.61 | 0.000 |
| ENSG00000234912 | hsa-miR-495-3p | SNHG20 | 0.61 | 0.000 |
| ENSG00000269821 | hsa-miR-148b-3p | KCNQ1OT1 | -0.53 | 0.005 |
| ENSG00000269821 | hsa-miR-152-3p | KCNQ1OT1 | 0.55 | 0.005 |
| ENSG00000269821 | hsa-miR-148a-3p | KCNQ1OT1 | 0.60 | 0.001 |
| ENSG00000269821 | hsa-miR-29c-3p | KCNQ1OT1 | 0.61 | 0.000 |
| ENSG00000269821 | hsa-miR-29b-3p | KCNQ1OT1 | 0.70 | 0.000 |
| ENSG00000269821 | hsa-miR-326 | KCNQ1OT1 | -0.51 | 0.009 |
| ENSG00000269821 | hsa-miR-330-5p | KCNQ1OT1 | 0.60 | 0.001 |
| ENSG00000269821 | hsa-miR-29a-3p | KCNQ1OT1 | 0.63 | 0.000 |
| ENSG00000269821 | hsa-miR-7-5p | KCNQ1OT1 | 0.60 | 0.001 |
| ENSG00000269821 | hsa-miR-140-5p | KCNQ1OT1 | 0.66 | 0.000 |
| ENSG00000269821 | hsa-miR-24-3p | KCNQ1OT1 | 0.65 | 0.000 |
| ENSG00000269821 | hsa-miR-335-5p | KCNQ1OT1 | 0.66 | 0.000 |
| ENSG00000244479 | hsa-miR-653-5p | OR2A1-AS1 | -0.60 | 0.000 |
| ENSG00000130600 | hsa-miR-130a-3p | H19 | 0.60 | 0.001 |
| ENSG00000130600 | hsa-miR-454-3p | H19 | 0.61 | 0.001 |
| ENSG00000130600 | hsa-miR-130b-3p | H19 | 0.65 | 0.000 |
| ENSG00000130600 | hsa-miR-29c-3p | H19 | 0.55 | 0.005 |
| ENSG00000130600 | hsa-miR-29b-3p | H19 | 0.54 | 0.007 |
| ENSG00000130600 | hsa-miR-29a-3p | H19 | 0.55 | 0.006 |
| ENSG00000130600 | hsa-miR-4295 | H19 | -0.65 | 0.000 |
| ENSG00000130600 | hsa-miR-3666 | H19 | -0.65 | 0.000 |
| ENSG00000130600 | hsa-miR-138-5p | H19 | -0.54 | 0.004 |
| ENSG00000130600 | hsa-miR-107 | H19 | 0.60 | 0.001 |
| ENSG00000130600 | hsa-miR-103a-3p | H19 | 0.68 | 0.000 |
| ENSG00000130600 | hsa-miR-370-3p | H19 | 0.61 | 0.000 |
| ENSG00000251562 | hsa-miR-124-3p | MALAT1 | 0.73 | 0.000 |
| ENSG00000251562 | hsa-miR-1271-5p | MALAT1 | 0.78 | 0.000 |
| ENSG00000251562 | hsa-miR-96-5p | MALAT1 | 0.56 | 0.004 |
| ENSG00000251562 | hsa-miR-506-3p | MALAT1 | 0.64 | 0.000 |
| ENSG00000251562 | hsa-miR-200b-3p | MALAT1 | -0.54 | 0.005 |
| ENSG00000251562 | hsa-miR-429 | MALAT1 | 0.64 | 0.000 |
| ENSG00000251562 | hsa-miR-200c-3p | MALAT1 | -0.85 | 0.000 |
| ENSG00000025772 | hsa-miR-326 | TOMM34 | -0.58 | 0.001 |
| ENSG00000025772 | hsa-miR-330-5p | TOMM34 | -0.54 | 0.005 |
| ENSG00000025772 | hsa-miR-421 | TOMM34 | 0.73 | 0.000 |
| ENSG00000079335 | hsa-miR-374b-5p | CDC14A | -0.68 | 0.000 |
| ENSG00000079335 | hsa-miR-374a-5p | CDC14A | -0.66 | 0.000 |
| ENSG00000065320 | hsa-miR-374b-5p | NTN1 | -0.67 | 0.000 |
| ENSG00000065320 | hsa-miR-374a-5p | NTN1 | -0.67 | 0.000 |
| ENSG00000198121 | hsa-miR-374b-5p | LPAR1 | -0.76 | 0.000 |
| ENSG00000198121 | hsa-miR-374a-5p | LPAR1 | -0.73 | 0.000 |
| ENSG00000156218 | hsa-miR-374b-5p | ADAMTSL3 | -0.57 | 0.001 |
| ENSG00000156218 | hsa-miR-374a-5p | ADAMTSL3 | -0.56 | 0.002 |
| ENSG00000164176 | hsa-miR-374b-5p | EDIL3 | -0.67 | 0.000 |
| ENSG00000164176 | hsa-miR-374a-5p | EDIL3 | -0.61 | 0.000 |
| ENSG00000220205 | hsa-miR-374b-5p | VAMP2 | -0.53 | 0.005 |
| ENSG00000220205 | hsa-miR-374a-5p | VAMP2 | -0.56 | 0.002 |
| ENSG00000066468 | hsa-miR-374b-5p | FGFR2 | -0.70 | 0.000 |
| ENSG00000066468 | hsa-miR-374a-5p | FGFR2 | -0.70 | 0.000 |
| ENSG00000065534 | hsa-miR-374b-5p | MYLK | -0.59 | 0.001 |
| ENSG00000065534 | hsa-miR-374a-5p | MYLK | -0.57 | 0.001 |
| ENSG00000143995 | hsa-miR-374b-5p | MEIS1 | -0.61 | 0.000 |
| ENSG00000143995 | hsa-miR-374a-5p | MEIS1 | -0.59 | 0.001 |
| ENSG00000198910 | hsa-miR-374b-5p | L1CAM | -0.51 | 0.009 |
| ENSG00000198910 | hsa-miR-374a-5p | L1CAM | -0.54 | 0.004 |
| ENSG00000119938 | hsa-miR-374b-5p | PPP1R3C | -0.64 | 0.000 |
| ENSG00000119938 | hsa-miR-374a-5p | PPP1R3C | -0.62 | 0.000 |
| ENSG00000137941 | hsa-miR-374b-5p | TTLL7 | -0.53 | 0.005 |
| ENSG00000137941 | hsa-miR-374a-5p | TTLL7 | -0.55 | 0.003 |
| ENSG00000126821 | hsa-miR-374b-5p | SGPP1 | -0.60 | 0.000 |
| ENSG00000126821 | hsa-miR-374a-5p | SGPP1 | -0.61 | 0.000 |
| ENSG00000111913 | hsa-miR-374b-5p | RIPOR2 | -0.59 | 0.001 |
| ENSG00000111913 | hsa-miR-374a-5p | RIPOR2 | -0.55 | 0.003 |
| ENSG00000169851 | hsa-miR-374b-5p | PCDH7 | -0.58 | 0.001 |
| ENSG00000169851 | hsa-miR-374a-5p | PCDH7 | -0.56 | 0.002 |
| ENSG00000111962 | hsa-miR-374b-5p | UST | -0.65 | 0.000 |
| ENSG00000111962 | hsa-miR-374a-5p | UST | -0.67 | 0.000 |
| ENSG00000134982 | hsa-miR-374b-5p | APC | -0.52 | 0.006 |
| ENSG00000134982 | hsa-miR-374a-5p | APC | -0.52 | 0.006 |
| ENSG00000143515 | hsa-miR-374b-5p | ATP8B2 | -0.61 | 0.000 |
| ENSG00000143515 | hsa-miR-374a-5p | ATP8B2 | -0.57 | 0.001 |
| ENSG00000137558 | hsa-miR-374b-5p | PI15 | -0.59 | 0.001 |
| ENSG00000137558 | hsa-miR-374a-5p | PI15 | -0.55 | 0.003 |
| ENSG00000129682 | hsa-miR-374b-5p | FGF13 | 0.57 | 0.003 |
| ENSG00000129682 | hsa-miR-374a-5p | FGF13 | -0.51 | 0.008 |
| ENSG00000163235 | hsa-miR-374b-5p | TGFA | -0.67 | 0.000 |
| ENSG00000163235 | hsa-miR-374a-5p | TGFA | -0.67 | 0.000 |
| ENSG00000197343 | hsa-miR-374b-5p | ZNF655 | -0.57 | 0.001 |
| ENSG00000197343 | hsa-miR-374a-5p | ZNF655 | -0.61 | 0.000 |
| ENSG00000114251 | hsa-miR-374b-5p | WNT5A | -0.55 | 0.003 |
| ENSG00000114251 | hsa-miR-374a-5p | WNT5A | 0.55 | 0.005 |
| ENSG00000155545 | hsa-miR-4295 | MIER3 | -0.66 | 0.000 |
| ENSG00000155545 | hsa-miR-130a-3p | MIER3 | -0.81 | 0.000 |
| ENSG00000155545 | hsa-miR-454-3p | MIER3 | -0.75 | 0.000 |
| ENSG00000155545 | hsa-miR-301a-3p | MIER3 | -0.83 | 0.000 |
| ENSG00000155545 | hsa-miR-301b-3p | MIER3 | -0.65 | 0.000 |
| ENSG00000155545 | hsa-miR-130b-3p | MIER3 | -0.61 | 0.000 |
| ENSG00000155545 | hsa-miR-3666 | MIER3 | -0.66 | 0.000 |
| ENSG00000105976 | hsa-miR-130a-3p | MET | -0.51 | 0.008 |
| ENSG00000105976 | hsa-miR-454-3p | MET | 0.68 | 0.000 |
| ENSG00000105976 | hsa-miR-301a-3p | MET | -0.52 | 0.006 |
| ENSG00000105976 | hsa-miR-301b-3p | MET | -0.53 | 0.005 |
| ENSG00000105976 | hsa-miR-130b-3p | MET | 0.67 | 0.000 |
| ENSG00000131389 | hsa-miR-130a-3p | SLC6A6 | 0.69 | 0.000 |
| ENSG00000131389 | hsa-miR-454-3p | SLC6A6 | 0.72 | 0.000 |
| ENSG00000131389 | hsa-miR-301a-3p | SLC6A6 | 0.55 | 0.005 |
| ENSG00000131389 | hsa-miR-301b-3p | SLC6A6 | -0.56 | 0.002 |
| ENSG00000131389 | hsa-miR-130b-3p | SLC6A6 | 0.62 | 0.000 |
| ENSG00000137449 | hsa-miR-4295 | CPEB2 | -0.80 | 0.000 |
| ENSG00000137449 | hsa-miR-130a-3p | CPEB2 | -0.72 | 0.000 |
| ENSG00000137449 | hsa-miR-454-3p | CPEB2 | -0.72 | 0.000 |
| ENSG00000137449 | hsa-miR-301a-3p | CPEB2 | -0.86 | 0.000 |
| ENSG00000137449 | hsa-miR-301b-3p | CPEB2 | -0.67 | 0.000 |
| ENSG00000137449 | hsa-miR-130b-3p | CPEB2 | -0.53 | 0.005 |
| ENSG00000137449 | hsa-miR-3666 | CPEB2 | -0.80 | 0.000 |
| ENSG00000152413 | hsa-miR-130a-3p | HOMER1 | -0.58 | 0.001 |
| ENSG00000152413 | hsa-miR-454-3p | HOMER1 | 0.63 | 0.000 |
| ENSG00000152413 | hsa-miR-301a-3p | HOMER1 | 0.56 | 0.004 |
| ENSG00000152413 | hsa-miR-301b-3p | HOMER1 | 0.56 | 0.004 |
| ENSG00000152413 | hsa-miR-130b-3p | HOMER1 | 0.83 | 0.000 |
| ENSG00000070961 | hsa-miR-130a-3p | ATP2B1 | -0.88 | 0.000 |
| ENSG00000070961 | hsa-miR-454-3p | ATP2B1 | -0.78 | 0.000 |
| ENSG00000070961 | hsa-miR-301a-3p | ATP2B1 | -0.81 | 0.000 |
| ENSG00000070961 | hsa-miR-301b-3p | ATP2B1 | -0.59 | 0.001 |
| ENSG00000070961 | hsa-miR-130b-3p | ATP2B1 | 0.53 | 0.008 |
| ENSG00000139163 | hsa-miR-130a-3p | ETNK1 | -0.86 | 0.000 |
| ENSG00000139163 | hsa-miR-454-3p | ETNK1 | -0.74 | 0.000 |
| ENSG00000139163 | hsa-miR-301a-3p | ETNK1 | -0.68 | 0.000 |
| ENSG00000139163 | hsa-miR-301b-3p | ETNK1 | -0.53 | 0.005 |
| ENSG00000139163 | hsa-miR-130b-3p | ETNK1 | 0.54 | 0.007 |
| ENSG00000135763 | hsa-miR-137 | URB2 | -0.57 | 0.001 |
| ENSG00000179041 | hsa-miR-485-5p | RRS1 | -0.58 | 0.001 |
| ENSG00000106344 | hsa-miR-495-3p | RBM28 | 0.65 | 0.000 |
| ENSG00000103257 | hsa-miR-148b-3p | SLC7A5 | 0.52 | 0.010 |
| ENSG00000103257 | hsa-miR-152-3p | SLC7A5 | 0.70 | 0.000 |
| ENSG00000103257 | hsa-miR-148a-3p | SLC7A5 | 0.59 | 0.001 |
| ENSG00000198720 | hsa-miR-29c-3p | ANKRD13B | 0.57 | 0.003 |
| ENSG00000198720 | hsa-miR-29b-3p | ANKRD13B | 0.80 | 0.000 |
| ENSG00000198720 | hsa-miR-326 | ANKRD13B | -0.63 | 0.000 |
| ENSG00000198720 | hsa-miR-330-5p | ANKRD13B | -0.55 | 0.002 |
| ENSG00000198720 | hsa-miR-29a-3p | ANKRD13B | 0.58 | 0.002 |
| ENSG00000119969 | hsa-miR-7-5p | HELLS | 0.85 | 0.000 |
| ENSG00000119969 | hsa-miR-140-5p | HELLS | 0.69 | 0.000 |
| ENSG00000119969 | hsa-miR-24-3p | HELLS | 0.61 | 0.000 |
| ENSG00000082512 | hsa-miR-29c-3p | TRAF5 | 0.67 | 0.000 |
| ENSG00000082512 | hsa-miR-29b-3p | TRAF5 | 0.79 | 0.000 |
| ENSG00000082512 | hsa-miR-29a-3p | TRAF5 | 0.81 | 0.000 |
| ENSG00000114270 | hsa-miR-29c-3p | COL7A1 | 0.63 | 0.000 |
| ENSG00000114270 | hsa-miR-29b-3p | COL7A1 | 0.53 | 0.008 |
| ENSG00000114270 | hsa-miR-29a-3p | COL7A1 | -0.53 | 0.006 |
| ENSG00000047634 | hsa-miR-29c-3p | SCML1 | -0.51 | 0.008 |
| ENSG00000047634 | hsa-miR-29b-3p | SCML1 | 0.69 | 0.000 |
| ENSG00000047634 | hsa-miR-24-3p | SCML1 | 0.62 | 0.000 |
| ENSG00000047634 | hsa-miR-29a-3p | SCML1 | 0.67 | 0.000 |
| ENSG00000142632 | hsa-miR-29c-3p | ARHGEF19 | -0.55 | 0.003 |
| ENSG00000142632 | hsa-miR-29b-3p | ARHGEF19 | 0.61 | 0.000 |
| ENSG00000142632 | hsa-miR-29a-3p | ARHGEF19 | 0.52 | 0.010 |
| ENSG00000097046 | hsa-miR-29c-3p | CDC7 | -0.53 | 0.005 |
| ENSG00000097046 | hsa-miR-29b-3p | CDC7 | 0.54 | 0.007 |
| ENSG00000097046 | hsa-miR-335-5p | CDC7 | -0.53 | 0.006 |
| ENSG00000097046 | hsa-miR-29a-3p | CDC7 | -0.51 | 0.008 |
| ENSG00000136108 | hsa-miR-653-5p | CKAP2 | -0.62 | 0.000 |
| ENSG00000101955 | hsa-miR-130a-3p | SRPX | 0.61 | 0.001 |
| ENSG00000101955 | hsa-miR-454-3p | SRPX | -0.84 | 0.000 |
| ENSG00000101955 | hsa-miR-130b-3p | SRPX | -0.80 | 0.000 |
| ENSG00000146122 | hsa-miR-29c-3p | DAAM2 | 0.68 | 0.000 |
| ENSG00000146122 | hsa-miR-29b-3p | DAAM2 | -0.88 | 0.000 |
| ENSG00000146122 | hsa-miR-29a-3p | DAAM2 | -0.54 | 0.004 |
| ENSG00000068650 | hsa-miR-4295 | ATP11A | -0.73 | 0.000 |
| ENSG00000068650 | hsa-miR-130a-3p | ATP11A | 0.55 | 0.005 |
| ENSG00000068650 | hsa-miR-454-3p | ATP11A | 0.72 | 0.000 |
| ENSG00000068650 | hsa-miR-130b-3p | ATP11A | 0.69 | 0.000 |
| ENSG00000068650 | hsa-miR-3666 | ATP11A | -0.73 | 0.000 |
| ENSG00000168077 | hsa-miR-130a-3p | SCARA3 | 0.65 | 0.000 |
| ENSG00000168077 | hsa-miR-130b-3p | SCARA3 | -0.75 | 0.000 |
| ENSG00000196411 | hsa-miR-4295 | EPHB4 | -0.67 | 0.000 |
| ENSG00000196411 | hsa-miR-130a-3p | EPHB4 | 0.53 | 0.008 |
| ENSG00000196411 | hsa-miR-454-3p | EPHB4 | 0.60 | 0.001 |
| ENSG00000196411 | hsa-miR-130b-3p | EPHB4 | 0.69 | 0.000 |
| ENSG00000196411 | hsa-miR-3666 | EPHB4 | -0.67 | 0.000 |
| ENSG00000136295 | hsa-miR-4295 | TTYH3 | -0.61 | 0.000 |
| ENSG00000136295 | hsa-miR-130a-3p | TTYH3 | 0.54 | 0.007 |
| ENSG00000136295 | hsa-miR-454-3p | TTYH3 | 0.56 | 0.004 |
| ENSG00000136295 | hsa-miR-130b-3p | TTYH3 | 0.56 | 0.004 |
| ENSG00000136295 | hsa-miR-3666 | TTYH3 | -0.61 | 0.000 |
| ENSG00000198720 | hsa-miR-138-5p | ANKRD13B | 0.53 | 0.008 |
| ENSG00000117385 | hsa-miR-29c-3p | P3H1 | 0.64 | 0.000 |
| ENSG00000117385 | hsa-miR-29b-3p | P3H1 | -0.53 | 0.005 |
| ENSG00000117385 | hsa-miR-29a-3p | P3H1 | -0.59 | 0.001 |
| ENSG00000103034 | hsa-miR-107 | NDRG4 | 0.60 | 0.001 |
| ENSG00000103034 | hsa-miR-103a-3p | NDRG4 | 0.64 | 0.000 |
| ENSG00000106089 | hsa-miR-29c-3p | STX1A | 0.69 | 0.000 |
| ENSG00000106089 | hsa-miR-29b-3p | STX1A | 0.75 | 0.000 |
| ENSG00000106089 | hsa-miR-29a-3p | STX1A | 0.68 | 0.000 |
| ENSG00000184371 | hsa-miR-4295 | CSF1 | -0.60 | 0.000 |
| ENSG00000184371 | hsa-miR-130a-3p | CSF1 | -0.53 | 0.006 |
| ENSG00000184371 | hsa-miR-454-3p | CSF1 | -0.79 | 0.000 |
| ENSG00000184371 | hsa-miR-130b-3p | CSF1 | -0.79 | 0.000 |
| ENSG00000184371 | hsa-miR-3666 | CSF1 | -0.60 | 0.000 |
| ENSG00000175592 | hsa-miR-130a-3p | FOSL1 | 0.58 | 0.002 |
| ENSG00000175592 | hsa-miR-138-5p | FOSL1 | -0.51 | 0.008 |
| ENSG00000175592 | hsa-miR-454-3p | FOSL1 | 0.64 | 0.000 |
| ENSG00000175592 | hsa-miR-130b-3p | FOSL1 | 0.68 | 0.000 |
| ENSG00000148516 | hsa-miR-4295 | ZEB1 | -0.83 | 0.000 |
| ENSG00000148516 | hsa-miR-130a-3p | ZEB1 | 0.63 | 0.000 |
| ENSG00000148516 | hsa-miR-454-3p | ZEB1 | -0.70 | 0.000 |
| ENSG00000148516 | hsa-miR-130b-3p | ZEB1 | -0.81 | 0.000 |
| ENSG00000148516 | hsa-miR-3666 | ZEB1 | -0.83 | 0.000 |
| ENSG00000108821 | hsa-miR-29c-3p | COL1A1 | 0.72 | 0.000 |
| ENSG00000108821 | hsa-miR-29b-3p | COL1A1 | -0.60 | 0.000 |
| ENSG00000108821 | hsa-miR-107 | COL1A1 | 0.67 | 0.000 |
| ENSG00000108821 | hsa-miR-103a-3p | COL1A1 | 0.67 | 0.000 |
| ENSG00000108821 | hsa-miR-29a-3p | COL1A1 | 0.52 | 0.010 |
| ENSG00000137558 | hsa-miR-29c-3p | PI15 | 0.62 | 0.000 |
| ENSG00000137558 | hsa-miR-29b-3p | PI15 | -0.73 | 0.000 |
| ENSG00000137558 | hsa-miR-29a-3p | PI15 | 0.57 | 0.003 |
| ENSG00000134013 | hsa-miR-29c-3p | LOXL2 | 0.67 | 0.000 |
| ENSG00000134013 | hsa-miR-29b-3p | LOXL2 | -0.59 | 0.000 |
| ENSG00000134013 | hsa-miR-29a-3p | LOXL2 | -0.58 | 0.001 |
| ENSG00000159167 | hsa-miR-4295 | STC1 | -0.76 | 0.000 |
| ENSG00000159167 | hsa-miR-130a-3p | STC1 | 0.53 | 0.008 |
| ENSG00000159167 | hsa-miR-454-3p | STC1 | 0.58 | 0.002 |
| ENSG00000159167 | hsa-miR-130b-3p | STC1 | 0.54 | 0.006 |
| ENSG00000159167 | hsa-miR-3666 | STC1 | -0.76 | 0.000 |
| ENSG00000134901 | hsa-miR-29c-3p | KDELC1 | 0.61 | 0.001 |
| ENSG00000134901 | hsa-miR-29b-3p | KDELC1 | -0.51 | 0.008 |
| ENSG00000134901 | hsa-miR-29a-3p | KDELC1 | -0.51 | 0.008 |
| ENSG00000124225 | hsa-miR-4295 | PMEPA1 | -0.66 | 0.000 |
| ENSG00000124225 | hsa-miR-130a-3p | PMEPA1 | 0.76 | 0.000 |
| ENSG00000124225 | hsa-miR-454-3p | PMEPA1 | 0.63 | 0.000 |
| ENSG00000124225 | hsa-miR-130b-3p | PMEPA1 | -0.55 | 0.002 |
| ENSG00000124225 | hsa-miR-3666 | PMEPA1 | -0.66 | 0.000 |
| ENSG00000177732 | hsa-miR-29c-3p | SOX12 | 0.56 | 0.004 |
| ENSG00000177732 | hsa-miR-29b-3p | SOX12 | 0.65 | 0.000 |
| ENSG00000177732 | hsa-miR-370-3p | SOX12 | -0.51 | 0.008 |
| ENSG00000177732 | hsa-miR-29a-3p | SOX12 | 0.55 | 0.005 |
| ENSG00000204262 | hsa-miR-29c-3p | COL5A2 | 0.73 | 0.000 |
| ENSG00000204262 | hsa-miR-29b-3p | COL5A2 | -0.65 | 0.000 |
| ENSG00000204262 | hsa-miR-29a-3p | COL5A2 | 0.52 | 0.010 |
| ENSG00000138411 | hsa-miR-4295 | HECW2 | -0.81 | 0.000 |
| ENSG00000138411 | hsa-miR-130a-3p | HECW2 | 0.52 | 0.009 |
| ENSG00000138411 | hsa-miR-454-3p | HECW2 | 0.52 | 0.009 |
| ENSG00000138411 | hsa-miR-130b-3p | HECW2 | 0.54 | 0.007 |
| ENSG00000138411 | hsa-miR-3666 | HECW2 | -0.81 | 0.000 |
| ENSG00000113140 | hsa-miR-29c-3p | SPARC | 0.68 | 0.000 |
| ENSG00000113140 | hsa-miR-29b-3p | SPARC | -0.63 | 0.000 |
| ENSG00000113140 | hsa-miR-29a-3p | SPARC | -0.55 | 0.003 |
| ENSG00000211448 | hsa-miR-29c-3p | DIO2 | 0.70 | 0.000 |
| ENSG00000211448 | hsa-miR-29b-3p | DIO2 | -0.64 | 0.000 |
| ENSG00000211448 | hsa-miR-29a-3p | DIO2 | -0.52 | 0.007 |
| ENSG00000066735 | hsa-miR-29c-3p | KIF26A | 0.54 | 0.006 |
| ENSG00000066735 | hsa-miR-29b-3p | KIF26A | -0.62 | 0.000 |
| ENSG00000066735 | hsa-miR-29a-3p | KIF26A | -0.53 | 0.005 |
| ENSG00000059588 | hsa-miR-124-3p | TARBP1 | -0.73 | 0.000 |
| ENSG00000059588 | hsa-miR-1271-5p | TARBP1 | -0.74 | 0.000 |
| ENSG00000059588 | hsa-miR-96-5p | TARBP1 | 0.71 | 0.000 |
| ENSG00000059588 | hsa-miR-506-3p | TARBP1 | -0.52 | 0.007 |
| ENSG00000157168 | hsa-miR-200b-3p | NRG1 | -0.61 | 0.000 |
| ENSG00000157168 | hsa-miR-429 | NRG1 | -0.64 | 0.000 |
| ENSG00000157168 | hsa-miR-200c-3p | NRG1 | -0.60 | 0.000 |
| ENSG00000182983 | hsa-miR-200b-3p | ZNF662 | -0.59 | 0.000 |
| ENSG00000182983 | hsa-miR-429 | ZNF662 | -0.69 | 0.000 |
| ENSG00000182983 | hsa-miR-200c-3p | ZNF662 | -0.60 | 0.000 |
