# Supplementary material for: Long non-coding RNA AC087388.1 as a novel biomarker in colorectal cancer
Source: BMC Cancer. 2022 Feb 21;22:196. doi: 10.1186/s12885-022-09282-0 (PMC8862536; doi:10.1186/s12885-022-09282-0)
Supplement: Supplementary file 4 — Additional file 4: Table S4. Comparisonof the TCGA gene expression with our patients. [file 12885_2022_9282_MOESM4_ESM.docx]

Table S4. Comparison of the TCGA gene expression with our patients.

|  | **Fold Change** | |
| --- | --- | --- |
| **LncRNA** | **TCGA** | **Our Patients** |
| **SLC16** | 1.48 | 1.13 |
| **SAT2** | 1.29 | 2.07 |
| **ELN1** | 4.53 | 4.03 |
